# The Impact of *KRAS* Mutation in Patients With Sporadic Nonampullary Duodenal Epithelial Tumors

**DOI:** 10.14309/ctg.0000000000000424

**Published:** 2021-11-18

**Authors:** Hideaki Kinugasa, Hiromitsu Kanzaki, Takehiro Tanaka, Shumpei Yamamoto, Yasushi Yamasaki, Kazuhiro Nouso, Kouichi Ichimura, Masahiro Nakagawa, Toshiharu Mitsuhashi, Hiroyuki Okada

**Affiliations:** 1 Department of Gastroenterology and Hepatology, Okayama University Graduate School of Medicine, Dentistry, and Pharmaceutical Sciences, Kita-ku, Okayama, Japan;; 2 Department of Pathology, Okayama University Graduate School of Medicine, Dentistry, and Pharmaceutical Sciences, Kita-ku, Okayama, Japan;; 3 Department of Pathology, Hiroshima City Hiroshima Citizens Hospital, Naka-ku, Hirosima, Japan;; 4 Department of Endoscopy, Hiroshima City Hiroshima Citizens Hospital, Naka-ku, Hirosima, Japan;; 5 Center for Innovative Clinical Medicine, Okayama University Hospital, Okayama, Japan.

## Abstract

**INTRODUCTION:**

The genomic characterization of primary nonampullary duodenal adenocarcinoma indicates a genetic resemblance to gastric and colorectal cancers. However, a correlation between the clinical and molecular characteristics of these cancers has not been established. This study aimed to elucidate the clinicopathological features of sporadic nonampullary duodenal epithelial tumors, including their molecular characteristics and prognostic factors.

**METHODS:**

One hundred forty-eight patients with sporadic nonampullary duodenal epithelial tumors were examined in this study. Patient sex, age, TNM stage, tumor location, treatment methods, histology, *KRAS* mutation, *BRAF* mutation, *Fusobacterium nucleatum*, mucin phenotype, and programmed death-ligand 1 (PD-L1) status were evaluated. *KRAS* and *BRAF* mutations, *Fusobacterium nucleatum*, mucin phenotype, and PD-L1 status were analyzed by direct sequencing, quantitative polymerase chain reaction, and immunochemical staining.

**RESULTS:**

The median follow-up duration was 119.4 months. There were no deaths from duodenal adenoma (the primary disease). Kaplan-Meier analysis for duodenal adenocarcinoma showed a significant effect of TNM stage (*P* < 0.01). In univariate analysis of primary deaths from duodenal adenocarcinoma, TNM stage II or higher, undifferentiated, *KRAS* mutations, gastric phenotype, intestinal phenotype, and PD-L1 status were significant factors. In multivariate analysis, TNM stage II or higher (hazard ratio: 1.63 × 10^10^, 95% confidence interval: 18.66–6.69 × 10^36^) and *KRAS* mutation (hazard ratio: 3.49, confidence interval: 1.52–7.91) were significant factors.

**DISCUSSION:**

Only *KRAS* mutation was a significant prognostic factor in primary sporadic nonampullary duodenal adenocarcinoma in cases in which TNM stage was considered.

## INTRODUCTION

In recent years, the clarification of gastrointestinal tumor characteristics has been enabled by instrumentation and reagent developments in endoscopy and DNA sequencing (1). In particular, the molecular biological characteristics of colorectal cancer have been determined, and the choice of treatment such as *EGFR* inhibitors, *BRAF* inhibitors, angiogenesis inhibitors, and immune checkpoint inhibitors differs in clinical practice depending on the presence or absence of *KRAS* mutation, *BRAF* mutation, and microsatellite instability (2). Tumor characteristics also contribute to prognosis, with anatomical location, such as the right or left side of the colon, identified as an important factor in determining treatment selection (3).

Sporadic nonampullary duodenal epithelial tumors (SNADETs) have been reported periodically. However, low detection rates compared with gastric and colorectal tumors have hampered efforts to analyze their clinicopathology. The prevalence of SNADETs is extremely low (0.02%–0.5%) (4–6), and primary sporadic nonampullary duodenal adenocarcinoma accounts for only 0.5% of all gastrointestinal malignancies (7). However, SNADET detection rates have improved with the development of endoscopic diagnosis techniques (8). Furthermore, SNADETs cases with a poor prognosis are on the rise (9). Consequently, the importance of research into SNADETs clinicopathology has been recognized, and their characteristics are becoming clearer as a result (10–12).

Whole-genome sequencing has revealed new insights into the genes involved in primary sporadic nonampullary duodenal adenocarcinoma (13). The genomic characterization of primary sporadic nonampullary duodenal adenocarcinoma indicates a genetic resemblance to gastric and colorectal cancers (13). However, a correlation between the clinical and molecular characteristics of these cancers has not been elucidated. On the other hand, the anatomical location of the tumor on the oral side of the papilla of Vater (oral side of Vater) (14,15), gastric mucin phenotype, which is one of the mucin phenotypes of the tumor (15,16), and programmed death-ligand 1 (PD-L1) status (17) are currently being investigated as factors potentially contributing to the prognosis of SNADETs. However, because of its rarity, the role of these factors has not been revealed in specific types of SNADETs, including duodenal adenoma and early- to advanced-stage duodenal adenocarcinoma.

In this study, prognostic factors were investigated in 148 patients with SNADETs. The analysis focused not only on tumor location, mucin phenotype, and PD-L1 status but also on factors such as *KRAS* and *BRAF* mutations, which are important in colorectal cancer. Furthermore, *Fusobacterium nucleatum* (*Fn*) (18), which has attracted research attention regarding its role in the progression of colorectal cancer, was also included in this investigation.

## METHODS

### Patients

One hundred forty-eight patients with SNADETs, treated at the Okayama University Hospital and Hiroshima City Hospital in Japan from 2006 to 2018, were enrolled in this study. Tissue samples were collected in all cases. Demographic, clinicopathological, and tumor characteristics were investigated and included patient sex, age, TNM stage, tumor location, treatment methods, histology, *KRAS* mutation, *BRAF* mutation, *Fn*, mucin phenotype, and PD-L1 status. The SNADETs were staged in accordance with the Union for International Cancer Control TNM staging system (19). In this study, stages 0 and Ⅰ were defined as early stage and stages II, III, and IV as advanced stage. The median follow-up duration was 47.7 months (range, 0.2–163 months). Somatic mutations of *KRAS* and *BRAF*, *Fn*, mucin phenotype, and PD-L1 status were examined by direct sequencing, real-time quantitative polymerase chain reaction (qPCR), and immunohistochemistry.

Endoscopic treatment was used for adenomas and duodenal adenocarcinoma (stage 0), surgery for duodenal cancer without distant metastasis (stage II and stage III), and chemotherapy for duodenal adenocarcinoma with distant metastasis (stage IV).

The Institutional Review Boards of Okayama University Hospital and Hiroshima City Hospital approved this study (2103-051/2021-8), which was conducted in accordance with the Declaration of Helsinki. Patients provided either written informed consent to participate or were required to opt out if their data were accessed retrospectively.

### DNA extraction

Formalin-fixed, paraffin-embedded (FFPE) tissue blocks were obtained from patients who had been biopsied, or resected by endoscopy or surgery, for SNADETs. All tissue sections were reviewed by expert gastrointestinal pathologists (T.T. and K.I.). Histological examinations confirmed that the samples contained a minimum of 30% tumor cells. DNA was extracted from five 10-μm-thick sections of the FFPE samples using a QIAamp DNA FFPE Tissue Kit (Qiagen, Valencia, CA), according to the manufacturer's instructions. All DNAs were eluted in a final volume of 50 μL and stored at −30 C. DNAs extracted from FFPE were quantified using a Qubit fluorometer (Thermo Fisher Scientific, Waltham, MA).

### Direct sequencing analysis

*KRAS* and *BRAF* were amplified using polymerase chain reaction (PCR) with forward and reverse primers (see Supplemental Table 1, Supplementary Digital Content 1, http://links.lww.com/CTG/A718). Each 50 μL PCR reaction contained 100 nM of each primer, 1 ng template DNA, and master mix reagent (AmpliTaq Gold 360 PCR Master Mix; Applied Biosystems, Foster City, CA). Amplification conditions consisted of 10 minutes at 94 C, followed by 40 cycles at 94 C for 10 seconds, 55 C for 30 seconds, and 72 C for 30 seconds, in a thermal cycler (GeneAmp PCR System 9700; Applied Biosystems). The PCR products were separated by electrophoresis on 2% agarose gels, stained with ethidium bromide, and visualized under ultraviolet light. Then, the PCR products were purified before direct sequencing was performed using the Big Dye Terminator Cycle Sequencing kit (Applied Biosystems) on an ABI Prism 310 genetic analyzer (Applied Biosystems). *KRAS* mutations in codons 12 and 13 and *BRAF* mutation in codon 600 were examined according to the raw nucleotide sequencing data in waveform obtained by direct sequencing.

### *Fn* analysis

The amount of *Fn* DNA in tissues was measured by qPCR. Custom-made primer/probe sets were used to amplify *Fn* and the reference human gene solute carrier organic anion transporter family number 2A1 (*SLCO2A1*), as previously described (20). The primer and probe sequences are summarized in Supplemental Table 1 (Supplementary Digital Content 1, http://links.lww.com/CTG/A718). The qPCR was performed in 20 μL reactions containing 30 ng of genomic DNA (2 μL), 1× final concentration Prime time gene expression Master Mix 2.0 (IDT, Coralville, IA) (10 μL), each Prime qPCR assay (FAM/HEX) (1 μL), and deionized distilled water (6 μL). The DNA was amplified and detected with a Roche LightCycler 96 system (Roche, Basel, Switzerland) under the following conditions: 10 minutes at 95 C, then 45 cycles of 15 seconds at 95 C and 60 seconds at 60 C.

All specimens were analyzed in duplicate. To exclude nonspecific PCR amplification, we regarded a specimen as *Fn* positive when both specimens were positive. The amount of *Fn* DNA in each tissue was calculated by 2−ΔCt, where ΔCt was the difference in the Ct value of *Fn* and *SLCO2A1*. The mean of the 2 Ct values for each reaction was used for analysis.

### Histopathological examinations and Vienna classification

For histological analysis, SNADETs tissue specimens were routinely fixed with formalin and completely embedded in paraffin. Tissue blocks were thinly sectioned, routinely processed, and stained with hematoxylin and eosin. All SNADETs were histologically graded based on the revised Vienna classification (VCL) system (21). We defined VCL category 3 as low-grade adenoma/dysplasia, 4.1 as high-grade adenoma/dysplasia, and 4.2 as carcinoma *in situ*. VCL categories 5.1 and 5.2 were considered as intramucosal carcinoma and submucosal carcinoma or beyond, respectively. We classified VCL categories 3 and 4.1 as adenoma and 4.2 or more as adenocarcinoma. Adenocarcinoma was subdivided into differentiated or undifferentiated types depending on histopathological grading. Two investigators (T.T. and K.I.) assessed histological grade independently, and any disagreements were resolved through consensus.

### Immunohistochemical examinations

FFPE tissue blocks were cut into 3-μm-thick tissue sections and subjected to hematoxylin and eosin and immunohistochemical staining. Immunohistochemical staining was performed by the standard avidin-biotin-peroxidase complex method with an automated immunostainer (BenchMark XT; Ventana Medical System, Tucson, AZ). Mucin phenotype of SNADETs was examined using MUC2 (Ccp58, monoclonal mouse; Dako, Denmark, UK), MUC5AC (CLH2, monoclonal mouse; Dako), MUC6 (CLH5, monoclonal mouse; Novus Biologicals, Littleton, CO), and CD10 (56C6, monoclonal mouse; Leica Biosystems, Newcastle, UK). The tumor's cytoplasmic immunoreactivity was judged positive for MUC2, MUC5AC, and MUC6. Luminal membranous immunoreactivity was judged positive for CD10. Immunohistochemical staining for gastric phenotype markers (MUC5AC and MUC6) and intestinal phenotype markers (MUC2 and CD10) were considered positive when distinct staining was observed in >10% of the cancer cells. The SNADETs were classified into 4 subtypes based on mucin immunohistochemistry: (1) gastric phenotype, (2) intestinal phenotype, (3) gastric and intestinal phenotype (mixed phenotype), and (4) not staining (null phenotype) (see Supplemental Figure 1, Supplementary Digital Content 1, http://links.lww.com/CTG/A717).

For PD-L1 staining, sections were retrieved in EDTA buffer (pH 8.0) at 98 C for 20 minutes. A monoclonal antibody was used against the membranous and cytoplasmic domain of PD-L1 (SP263, monoclonal rabbit; Ventana Medical Systems) in immunostaining, and reactivity was evaluated for cancer cells. PD-L1 positivity was defined as a positive cell staining of any intensity on ≥1% of the cell membrane and cytoplasm.

Two investigators (T.T. and K.I.), blinded to the patients' clinical information, collaboratively assessed the immunohistochemical results as well as the histological analysis and VCL classification.

### Statistical analysis

All continuous variables are reported as the median (range), and comparisons were made using the Wilcoxon rank-sum test. All categorical variables are summarized as frequencies (percentages), with Pearson χ^2^ or Fisher exact tests used for examining comparisons. Overall survival (OS) was estimated by the Kaplan-Meier method, and differences were evaluated using the log-rank test. A Cox proportional hazard model was used to assess OS by TNM stage, tumor location, treatment methods, *KRAS* mutation, *BRAF* mutation, *Fn*, mucin phenotype, and PD-L1 status. All statistical tests were 2 sided, and a *P* value less than 0.05 was considered statistically significant. Statistical analyses were performed using the JMP 14 software program (SAS Institute, Cary, NC).

## RESULTS

### Patient characteristics

Of the 148 patients with SNADETs, 55 and 93 had duodenal adenomas and duodenal adenocarcinomas, respectively. The median age was 67 years, and there were 97 men and 51 women. Tumors were located on the oral (n = 79) or anal (n = 69) side of the papilla of Vater. Some patients also received endoscopic treatment (n = 71), surgical procedures (n = 59), chemotherapy (n = 9), and other treatments (n = 9; Table 1).

**Table 1.** Patient and tumor characteristics

	Neoplasia (n = 148)		*P* value	Adenocarcinoma (n = 93)		*P* value
	Adenoma (n = 55)	Adenocarcinoma (n = 93)		Stages 0 and I (n = 45)	Stages II, III, and IV (n = 48)	
Sex (male/female)	36/19	61/32	0.98	28/17	33/15	0.50
Age, median (range)	65 (36–83)	68 (29–90)	0.33	68 (36–84)	68 (29–90)	0.54
VCL (3/4.1/4.2/5.1/5.2)	52/3/0/0/0	0/0/30/8/55	—	0/0/30/8/7	0/0/0/0/48	—
TNM stage (0/I/II/III/IV)	—	30/15/11/16/21	—	30/15/0/0/0	0/0/11/16/21	—
Location (oral Vater/anal Vater)	20/35	59/34	<0.01^a^	24/21	35/13	0.04^a^
Treatment (endoscopy/surgery/chemo/others)	50/5/0/0	21/54/9/9	<0.01^a^	21/24/0/0	0/30/9/9	<0.01^a^
Histology (differentiated/undifferentiated)	—	81/12	—	44/1	37/11	<0.01^a^
*KRAS*, n (%)	10 (18.1)	23 (24.7)	0.34	8 (17.7)	15 (31.2)	0.12
*BRAF*, n (%)	1 (1.8)	12 (12.9)	0.01^a^	2 (4.4)	10 (20.8)	0.01^a^
*Fusobacterium*, n (%)	6 (10.9)	23 (24.7)	0.03^a^	11 (24.4)	12 (25.0)	0.95
Mucin phenotype (gastric/intestinal/mix/null)	2/40/12/1	39/34/12/8	<0.01^a^	11/26/8/0	28/8/4/8	<0.01^a^
PD-L1 (negative/positive)	—	68/25	—	41/4	27/21	<0.01^a^

Chemo, chemotherapy; PD-L1, programmed death-ligand 1; VCL, Vienna classification.

a Statistically significant difference.

**Figure 1.**
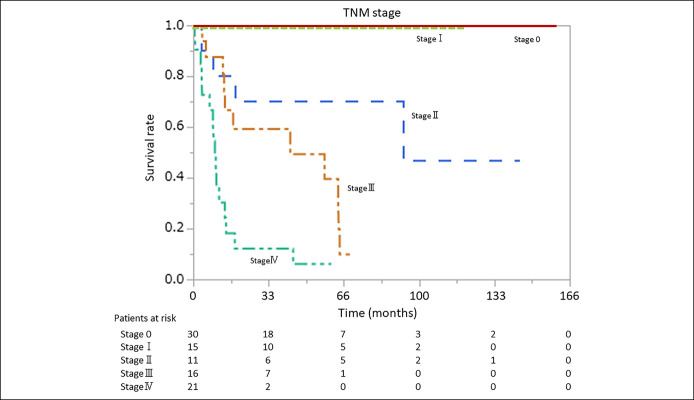
Kaplan-Meier plots for duodenal adenocarcinoma with TNM stage. There were no cases of primary mortality from duodenal adenocarcinoma in stages 0 and I. Median survival times in stages II, III, and IV were 93.1 months, 42.9 months, and 9.5 months, respectively (*P* < 0.01). This figure indicates disease specific survival.

There were no cases of primary mortality from duodenal adenoma. On the other hand, primary mortality from duodenal adenocarcinoma increased with TNM stage, involving 0, 0, 4, 11, and 16 patient deaths from stages 0, I, II, III, and IV, respectively. Kaplan-Meier analysis divided on TNM stage revealed a significant effect on OS, as median survival was 93.1 months in stage II, 42.9 months in stage III, and 9.5 months in stage IV (*P* < 0.01; Figure 1).

### Genetic and epigenetic characteristics

Of the 148 patients with SNADETs, 33 had *KRAS* mutation, 13 had *BRAF* mutation, and 29 were *Fn* positive. Furthermore, a range of patients demonstrated gastric phenotype (n = 41), intestinal phenotype (n = 74), mixed phenotype (n = 24), and null phenotype (n = 9; Table 1).

Comparing the 55 patients with duodenal adenoma with the 93 patients with duodenal adenocarcinoma showed significant differences in anatomical location (*P* < 0.01), treatment methods (*P* < 0.01), *BRAF* mutation (*P* = 0.01), *Fn* (*P* = 0.03), and mucin phenotype (*P* < 0.01). On the other hand, the 45 patients with early-stage duodenal adenocarcinoma showed significant differences in anatomical location (*P* = 0.04), treatment methods (*P* < 0.01), tumor histology (*P* < 0.01), *BRAF* mutation (*P* = 0.01), mucin phenotype (*P* < 0.01), and PD-L1 status (*P* < 0.01) compared with the 48 patients with advanced-stage duodenal adenocarcinoma (Table 1).

### Duodenal adenocarcinoma

**Figure 2.**
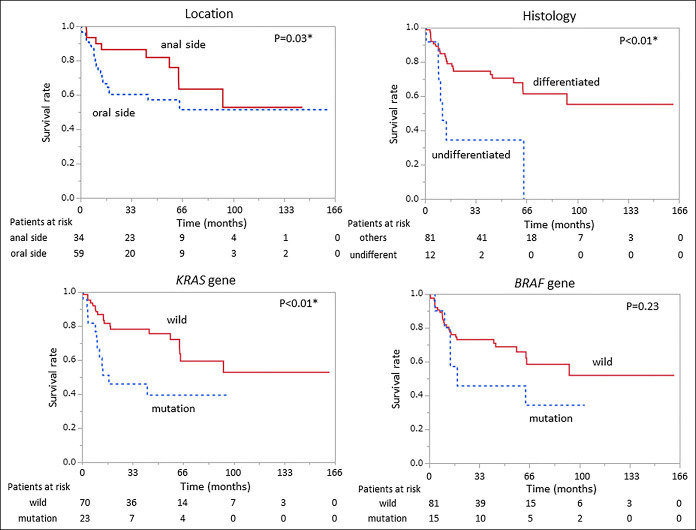
Kaplan-Meier plots for duodenal adenocarcinoma with location, histology, *KRAS*, and *BRAF*. Kaplan-Meier analysis of anatomical location (oral side of the papilla of Vater), histology (undifferentiated), *KRAS* mutation, and *BRAF* mutation showed that anatomical location (oral side of Vater; *P* = 0.03), undifferentiated (*P* < 0.01), and *KRAS* mutation (*P* < 0.01) had significant effects on overall survival.

**Figure 3.**
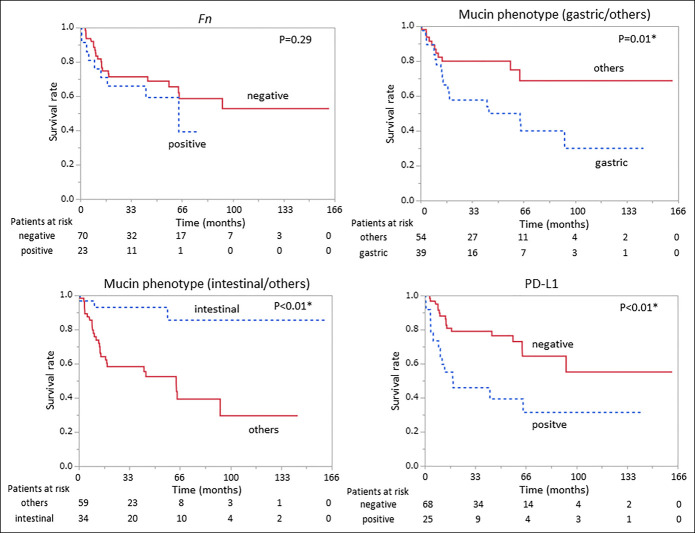
Kaplan-Meier plots for duodenal adenocarcinoma with *Fn*, mucin phenotype, and PD-L1 status. Kaplan-Meier analysis of *Fn*, mucin phenotype, and PD-L1 status showed that gastric phenotype (*P* = 0.01), intestinal phenotype (*P* < 0.01), and PD-L1 status (*P* < 0.01) had significant effects on overall survival. *Fn*, *Fusobacterium nucleatum*; PD-L1, programmed death-ligand 1.

Kaplan-Meier analysis of survival in duodenal adenocarcinoma showed that TNM stage II or higher (*P* < 0.01), anatomical location (oral side of Vater; *P* = 0.03), undifferentiated (*P* < 0.01), *KRAS* mutation (*P* < 0.01), gastric phenotype (*P* = 0.01), intestinal phenotype (*P* < 0.01), and PD-L1 status (*P* < 0.01) significantly influenced patient outcomes (Figures 1–3).

In univariate analysis of primary mortality from duodenal adenocarcinoma, TNM stage II or higher (hazard ratio [HR]: 1.8 × 10^10^, 95% confidence interval [CI]: not calculable; *P* < 0.01), undifferentiated (HR: 3.66, CI: 1.43–8.24; *P* < 0.01), *KRAS* mutation (HR: 2.44, CI: 1.14–4.99; *P* = 0.02), gastric phenotype (HR: 2.45, CI: 1.19–5.30; *P* = 0.01), intestinal phenotype (HR: 0.15, CI: 0.03–0.43; *P* < 0.01), and PD-L1 status (HR: 2.84, CI:1.37–5.77; *P* < 0.01) were significant factors (Table 2). In multivariate analysis, TNM stage II or higher (HR: 1.63 × 10^10^, CI: 18.66–6.69 × 10^36^; *P* < 0.01) and *KRAS* mutation (HR: 3.49, CI: 1.52–7.91; *P* < 0.01) were found to be significant factors (Table 2).

**Table 2.** Univariate and multivariate analyses of primary mortality from duodenal adenocarcinoma

	Univariate		Multivariate	
	HR	*P* value	HR	*P* value
TNM stage (II, III, IV/0I)	1.80 × 10^10^ (NA)	<0.01^a^	1.63 × 10^10^ (18.66–6.69 × 10^36^)	<0.01^a^
Location (anal Vater/oral Vater)	0.53 (0.23–1.13)	0.10		
Histology (undifferentiated/differentiated)	3.66 (1.43–8.24)	<0.01^a^	1.41 (0.43–4.24)	0.54
*KRAS* (mutation/wild)	2.44 (1.14–4.99)	0.02^a^	3.49 (1.52–7.91)	<0.01^a^
*BRAF* (mutation/wild)	1.71 (0.63–3.91)	0.26		
*Fusobacterium* (positive/negative)	1.51 (0.65–3.21)	0.31		
Mucin phenotype (gastric/others)	2.45 (1.19–5.30)	0.01^a^	0.58 (0.22–1.64)	0.29
Mucin phenotype (intestinal/others)	0.15 (0.03–0.43)	<0.01^a^	0.24 (0.04–1.05)	0.05
Mucin phenotype (mix/others)	0.92 (0.22–2.63)	0.90		
Mucin phenotype (null/others)	2.76 (0.92–6.74)	0.06		
PD-L1 (positive/negative)	2.84 (1.37–5.77)	<0.01^a^	1.02 (0.43–2.37)	0.95

NA: calculation was not possible due to the absence of primary deaths in stages 0 and I.

HR, hazard ratio; NA, not available; PD-L1, programmed death-ligand 1.

a Statistically significant difference.

### Characteristics of the patients with *KRAS* mutation

Patients with (n = 33) and without (n = 115) *KRAS* mutation exhibited significant differences in sex (*P* = 0.02), *BRAF* mutation (*P* < 0.01), and mucin phenotype (*P* < 0.01; Table 3). Moreover, of the 55 patients with duodenal adenoma, there were 10 patients with *KRAS* mutation, but no significant differences were found between these patients and the other 45 patients without *KRAS* mutation. However, among the 93 patients with duodenal adenocarcinoma, there were 23 patients with *KRAS* mutation, and they were significantly different from the 70 patients without *KRAS* mutation in terms of sex (*P* = 0.01), *BRAF* mutation (*P* < 0.01), and mucin phenotype (*P* = 0.01; Table 3).

**Table 3.** Clinicopathological features focusing on the *KRAS* gene

	Neoplasia (n = 148)		*P* value	Adenoma (n = 55)		*P* value	Adenocarcinoma (n = 93)		*P* value
	*KRAS* negative (n = 115)	*KRAS* positive (n = 33)		*KRAS* negative (n = 45)	*KRAS* positive (n = 10)		*KRAS* negative (n = 70)	*KRAS* positive (n = 23)	
Sex (male/female)	81/34	16/17	0.02^a^	30/15	6/4	0.69	51/19	10/13	0.01^a^
Age, median (range)	67 (29–84)	68 (46–90)	0.18	65 (36–83)	67.5 (46–80)	0.85	67 (29–84)	69 (51–90)	0.16
Tumor (adenoma/adenocarcinoma)	45/70	10/23	0.34	—	—	—	—	—	—
TNM stage (0/I/II/III/IV)	—	—	—	—	—	—	25/12/8/10/15	5/3/3/6/6	0.59
Location (oral Vater/anal Vater)	57/58	22/11	0.07	14/31	6/4	0.09	43/27	16/7	0.47
Treatment (endoscopy/surgery/chemo/others)	57/46/6/6	14/13/3/3	0.69	41/4/0/0	9/1/0/0	0.91	16/42/6/6	5/12/3/3	0.83
Histology (differentiated/undifferentiated)	—	—	—	—	—	—	60/10	21/2	0.47
*KRAS*, n (%)	—	—	—	—	—	—	—	—	—
*BRAF*, n (%)	13 (11.3)	0 (0)	<0.01^a^	1 (2.2)	0 (0)	0.52	12 (17.1)	0 (0)	<0.01^a^
*Fusobacterium*, n (%)	21 (18.2)	8 (24.2)	0.45	5 (11.1)	1 (10.0)	0.91	16 (22.8)	7 (30.4)	0.47
Mucin phenotype (gastric/intestinal/mix/null)	24/65/18/8	17/9/6/1	<0.01^a^	1/36/7/1	1/4/5/0	0.06	23/29/11/7	16/5/1/1	0.01^a^
PD-L1 (negative/positive)	—	—	—	—	—	—	52/18	16/7	0.66

PD-L1, programmed death-ligand 1.

a Statistically significant difference.

## DISCUSSION

We analyzed prognostic factors in 148 patients with SNADETs, focusing on TNM stage, the anatomical location of the tumor, *KRAS* mutation, *BRAF* mutation, *Fn*, mucin phenotype, and PD-L1 status. There were no primary deaths from nonampullary duodenal adenoma in this study. *KRAS* mutation was an independent factor for primary mortality in nonampullary duodenal adenocarcinoma, regardless of TNM stage (stage II or higher). These results indicate that *KRAS* mutation is a more important prognostic factor than anatomical location (oral side of Vater), gastric phenotype, and PD-L1 status, which have previously been reported as poor prognostic factors in sporadic nonampullary duodenal adenocarcinoma (14–16).

In the present study, anatomical location, mucin phenotype, and PD-L1 were significant factors influencing OS (Kaplan-Meier analysis), but its effects were not supported in multivariate analysis. These findings demonstrate that careful consideration should be given to a patient's background information when evaluating mucin phenotype and PD-L1 status as prognostic factors. However, there seems to be some correlation between higher TNM stage and mucin phenotype and PD-L1 status (Table 1), suggesting that early detection may be difficult due to rapid clinical progression. The high prevalence of PD-L1 in more advanced TNM stages suggests that a more personalized treatment strategy, such as immune checkpoint inhibitors, could be possible for advanced-stage duodenal adenocarcinoma.

With regard to the importance of *KRAS* mutations, there have been no previous reports showing a relationship between *KRAS* mutation and prognosis. Although some studies have reported subanalyses, all of which might have lacked significance because of the small number of patients included and the distribution of TNM stages (17,22). In our study, *KRAS* mutation was an independent prognostic factor along with TNM stage (stage II or higher), unlike anatomical location, mucinous phenotype, and PD-L1 status. Moreover, even if stages II, III, and IV were analyzed separately, *KRAS* mutation remained the only significant factor in stages II and III. Considering the poor prognosis of stage IV, the importance of *KRAS* mutation as a prognostic factor in stages II and III is even more distinguished (HR: 4.01 × 10^9^, CI: 2.70–4.90 × 10^308^; *P* = 0.01; HR: 17.04, CI: 2.01–433.72; *P* < 0.01). Also, it is very interesting to note from Table 3 that there is a relationship between *KRAS* and sex or gastric phenotype. However, *KRAS* and *BRAF* could be understood from the perspective of a paradoxical relationship.

The incidence of *KRAS* mutation in duodenal adenoma, early-stage duodenal adenocarcinoma, and advanced-stage duodenal adenocarcinoma was 18.1%, 17.7%, and 31.2%, respectively, with no significant differences (*P* = 0.20). There were cases of duodenal adenoma and early-stage duodenal adenocarcinoma with *KRAS* mutation but without primary mortality. Therefore, *KRAS* mutation in advanced-stage duodenal adenocarcinoma might have specific implications, and active treatment might be important for preventing advanced-stage neoplasia.

Relationships between the role of *KRAS* mutation and prognosis have been reported in various carcinomas such as colorectal cancer (23,24) and pancreatic cancer (25). Among the *KRAS* mutations, mutations in codon 12 and codon 13 are particularly noteworthy. Some basic studies have shown that the basic GTPase activity of G12V is about one-fourth that of G12D and one-tenth that of wild-type *KRAS* (26,27). Furthermore, Rat-1 cells with G12V mutations have been shown to be significantly more invasive *in vitro* than clones with G12D mutations or wild-type *KRAS* (28,29). Based on these results, it is easy to speculate that cells with G12V mutations are more invasive and contribute to a worse prognosis. In our current results, we could not find any significant difference between G12V and other type of *KRAS* mutation in relation to prognosis (*P* = 0.20), although median survival time in G12V was shorter than others (11 months and 31.5 months, respectively). To clarify which type of *KRAS* mutation is particularly associated with prognosis, we will continue to accumulate more cases.

The importance of *Fn* in colorectal tumors has recently been revealed. We have previously reported the presence of *Fn* in colorectal adenoma and colorectal cancer (20), and we hypothesized that *Fn* might play an important role in SNADETs as well. The incidence of *Fn* in duodenal adenoma, early-stage duodenal adenocarcinoma, and advanced-stage duodenal adenocarcinoma was 10.9%, 24.4%, and 25.0%, respectively (*P* = 0.10). However, *Fn* was not found to be a prognostic factor in this study.

Several limitations of the present study should be noted. First, this was a retrospective study, which may not provide the same level of evidence that could be achieved with a prospective study. Furthermore, comparisons based on other demographic factors, such as race, were not possible. It is hoped that international collaborative studies will enable the collection of more cases for investigation. Second, the prognosis shown in Kaplan-Meier analysis requires careful interpretation because sample size of this study is inevitable small due to the focus on rare disease. Third, although some previous study demonstrated higher risk of malignant transformation in duodenal adenocarcinoma with CpG island methylator phenotype (30), current study did not evaluate CpG island methylation.

Although conventional endoscopic treatment for duodenal adenomas and early-stage duodenal adenocarcinomas may be acceptable, the present findings suggest that treatment strategies for advanced-stage duodenal adenocarcinomas could potentially undergo a major shift. In light of our new findings, it is likely that molecularly targeted therapies, such as *KRAS* inhibitors, *BRAF* inhibitors, angiogenesis inhibitors, and immune checkpoint inhibitors, should be validated for treating advanced-stage duodenal adenocarcinoma, similar to the studies of colorectal cancer. If the response of duodenal adenocarcinoma was similar to that of colorectal cancer, angiogenesis inhibitors would be better than *EGFR* inhibitors for the treatment of duodenal adenocarcinoma with *KRAS* mutation. *KRAS* status might also be useful when considering adjuvant or neoadjuvant chemotherapy for duodenal adenocarcinoma, although the effect is still unclear at present. Also, liquid biopsy, which has been attracting attention as a noninvasive method in recent years (31), could be used to monitor treatment resistance in duodenal adenocarcinoma by targeting *KRAS* mutations.

In conclusion, *KRAS* mutation was found to be a significant independent factor for prognosis, in addition to TNM stage. Although further molecular biological analysis is required to investigate the usefulness of candidate genes other than *KRAS* and *BRAF*, assessing *KRAS* mutation could be a very useful tool for treating sporadic nonampullary duodenal adenocarcinoma.

## CONFLICTS OF INTEREST

**Guarantor of the article:** Hideaki Kinugasa, MD, PhD.

**Specific author contributions:** H. Kinugasa designed and drafted the manuscript. H. Kinugasa, Y.Y., and M.N. collected the clinical data. H. Kinugasa, H. Kanzaki, T.T., S.Y., K.I., and T.M. were responsible for experiments. H. Kinugasa and T.M. analyzed the data. H. Kinugasa, K.N., and H.O. supervised the manuscript preparation. All authors approved the final manuscript.

**Financial support:** This study was supported by JSPS KAKENHI (19k17433).

**Potential competing interests:** None to report. This study was approved by the Institutional Review Boards of Okayama University Hospital and Hiroshima City Hospital and conducted in accordance with the Declaration of Helsinki.

**Study Highlights**

**WHAT IS KNOWN**

✓ Sporadic nonampullary duodenal epithelial tumor detection rates are rising; however, their clinicopathology is poorly documented.

**WHAT IS NEW HERE**

✓ Sporadic nonampullary duodenal adenoma and early-stage adenocarcinoma had better survival.
✓ *KRAS* mutation of sporadic nonampullary duodenal adenocarcinoma was a significant prognostic factor.

## Supplementary Material

SUPPLEMENTARY MATERIAL
