# Supplementary material for: The Impact of KRAS Mutation in Patients With Sporadic Nonampullary Duodenal Epithelial Tumors
Source: Clin Transl Gastroenterol. 2021 Nov 18;12(11):e00424. doi: 10.14309/ctg.0000000000000424 (PMC8604005; doi:10.14309/ctg.0000000000000424)
Supplement: SUPPLEMENTARY MATERIAL [file ct9-12-e00424-s001.docx]

**Supplemental Table 1. Primer and probe sequences.**

| Primer/ Probe | Sequences (5’-3’) |
| --- | --- |
| *KRAS* forward primer | 5ʹ-AACCTTATGTGTGACATGTTCTAATAT-3ʹ |
| *KRAS* reverse primer | 5ʹ-TCGTCCACAAAATGATTCTGAATT-3ʹ |
| *BRAF* forward primer | 5ʹ-CTACTGTTTTCCTTTACTTACTACACCTCAGA-3ʹ |
| *BRAF* reverse primer | 5ʹ-ATCCAGACAACTGTTCAAACTGATG-3ʹ |
| *Fn* forward primer | 5ʹ-CAACCATTACTTTAACTCTACCATGTTCA-3ʹ |
| *Fn* reverse primer | 5ʹ-GTTGACTTTACAGAAGGAGATTATGTAAAAATC-3ʹ |
| *Fn* probe | 5ʹ-GTTGACTTTACAGAAGGAGATTA-3ʹ |
| *SLCO2A1* forward primer | 5ʹ-ATCCCCAAAGCACCTGGTTT-3ʹ |
| *SLCO2A1* reverse primer | 5ʹ-AGAGGCCAAGATAGTCCTGGTAA-3ʹ |
| *SLCO2A1* probe | 5ʹ-CCATCCATGTCCTCATCT-3ʹ |
