# Supplementary figures and images for: The Impact of KRAS Mutation in Patients With Sporadic Nonampullary Duodenal Epithelial Tumors
Source: Clin Transl Gastroenterol. 2021 Nov 18;12(11):e00424. doi: 10.14309/ctg.0000000000000424 (PMC8604005; doi:10.14309/ctg.0000000000000424)

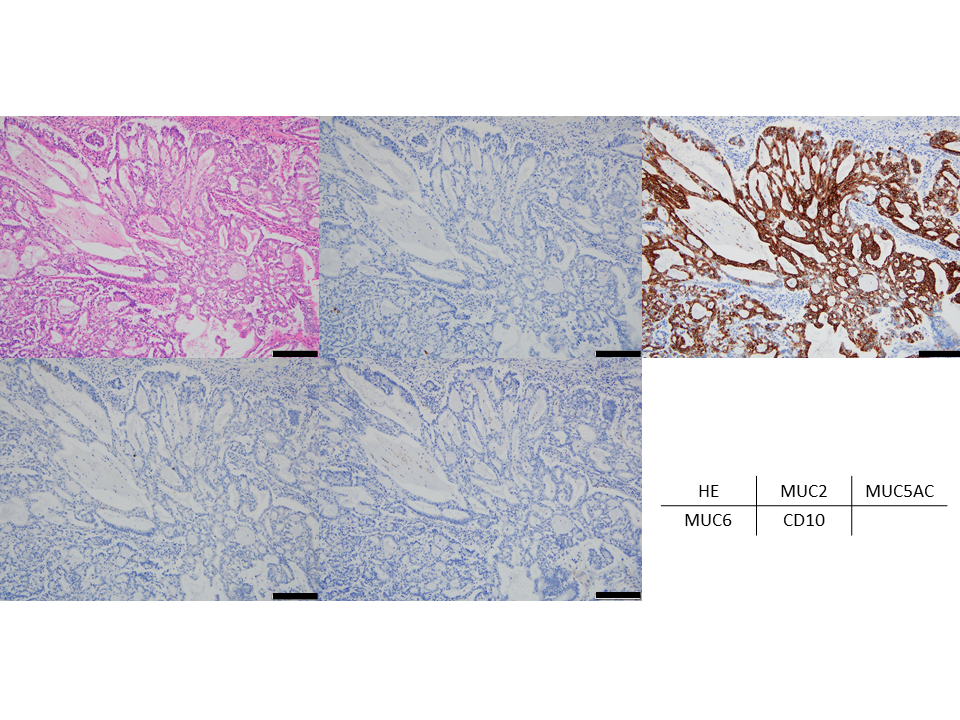

Supplement: SUPPLEMENTARY MATERIAL [file ct9-12-e00424-s002.tif]

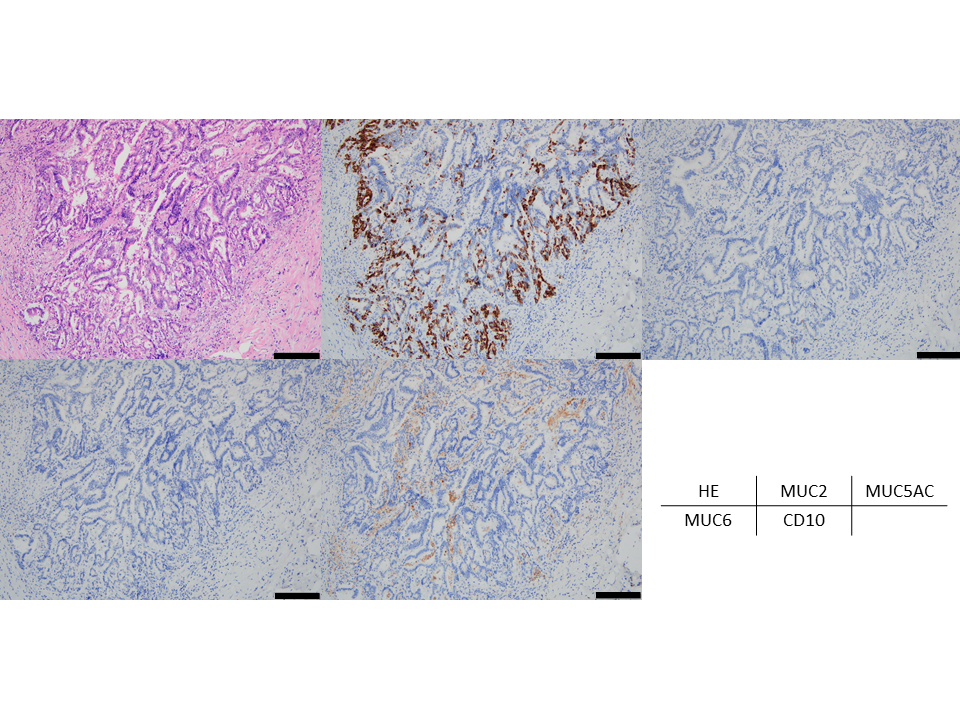

Supplement: SUPPLEMENTARY MATERIAL [file ct9-12-e00424-s003.tif]
