# Supplementary material for: The Impact of KRAS Mutation in Patients With Sporadic Nonampullary Duodenal Epithelial Tumors
Source: Clin Transl Gastroenterol. 2021 Nov 18;12(11):e00424. doi: 10.14309/ctg.0000000000000424 (PMC8604005; doi:10.14309/ctg.0000000000000424)
Supplement: SUPPLEMENTARY MATERIAL [file ct9-12-e00424-s004.docx]

**Supplemental Figure 1. Immunohistochemistry for mucin phenotype in non-ampullary duodenal adenocarcinoma.**

Representative gastric phenotype (A) and intestinal phenotype (B) in non-ampullary duodenal adenocarcinoma with HE (10×), MUC2 (10×), MUC5AC (10×), MUC6 (10×) and CD10 (10×). Bars: 200 μm.
